# T‐cell‐related skin inflammatory flareups with Th1 polarity in a patient with pseudoxanthoma elasticum

**DOI:** 10.1002/ski2.430

**Published:** 2024-11-26

**Authors:** Samuel Rocour, Emeline Vinatier, Céline Fassot, Jonathan Dauvé, Agnès Toutain, Sabrina Fronteau, Marine Monnier, Laurence Preisser, Anne Croué, Olivier Le Saux, Alain Morel, Yves Delneste, Ludovic Martin

**Affiliations:** ^1^ PXE National Reference Center MAGEC Nord University Hospital of Angers Angers France; ^2^ Immunology and Allergology Laboratory University Hospital of Angers Angers France; ^3^ University of Angers Nantes Université Inserm CNRS SFR ICAT LabEx IGO CRCI2NA Angers France; ^4^ University of Angers CNRS Inserm SFR ICAT Mitovasc Angers France; ^5^ ICO Cancer Center Angers France; ^6^ Pathology Department University Hospital of Angers Angers France; ^7^ Department of Cell and Molecular Biology John A. Burns School of Medicine University of Hawaii at Manoa Honolulu Hawaii USA

## Abstract

Pseudoxanthoma elasticum (PXE) is a genetic disorder characterized by ectopic calcification of tissues rich in elastic fibres (OMIM 264800). To date, PXE is considered a metabolic disease linked to an imbalance between pro‐ and anti‐calcifying factors. The occurrence of sporadic erythematous flareups of PXE skin lesions is a complaint that we heard about on several occasions at the French PXE reference centre. However, this rare clinical aspect had never been extensively studied. We have had the opportunity to investigate a 13‐year‐old patient experiencing an erythematous flareup of his PXE lesions. We conducted this work to identify what type of inflammation was implicated in his lesions. An incisional skin biopsy on a recent erythematous inguinal PXE lesion was performed and sent for histological and transcriptomic analyses. The findings were compared to a non‐erythematous PXE‐affected skin biopsy obtained from another young PXE patient. Histological examination revealed perivascular T‐cell infiltrates with Th1 polarity and elevated expression of cytotoxicity markers in RNAseq and RT‐qPCR analyses. There was no increase in Th17 or Th2 markers. Our findings support the previous evidence of a possible inflammatory component in the development of PXE. Whether Th1‐dependent inflammation contributes to the pathology as an active process or is an aggravating factor requires further investigations.

## Graphical abstract

We investigated a young PXE patient experiencing erythematous flares of his PXE lesions compared to another young PXE patient. Our results suggest that inflammation could be part of PXE pathophysiology.

## 1 INTRODUCTION

Pseudoxanthoma elasticum (PXE) is an inherited connective tissue disorder characterized by progressive calcification and fragmentation of elastic fibres in the mid‐dermis, Bruch's membrane of the retina and mid‐sized arteries. To date, PXE is considered a disease of ectopic calcification caused by inactivating variants in the *ABCC6* gene. *ABCC6* encodes a transporter primarily expressed in the liver and kidneys, whose substrate is still unknown.^1^ Recent evidence suggests an inflammatory component in PXE, supported by our observations of increased ^18^F‐FDG uptake and altered microvascular blood flow in affected areas.^2^, ^3^, ^4^ This letter highlights new insights into the inflammatory aspects of PXE. Indeed, we had the opportunity to investigate a young patient presenting with erythematous flares on his skin lesions, a manifestation rarely observed in PXE patients.

## 2 PATIENTS AND METHODS

**FIGURE 1:**
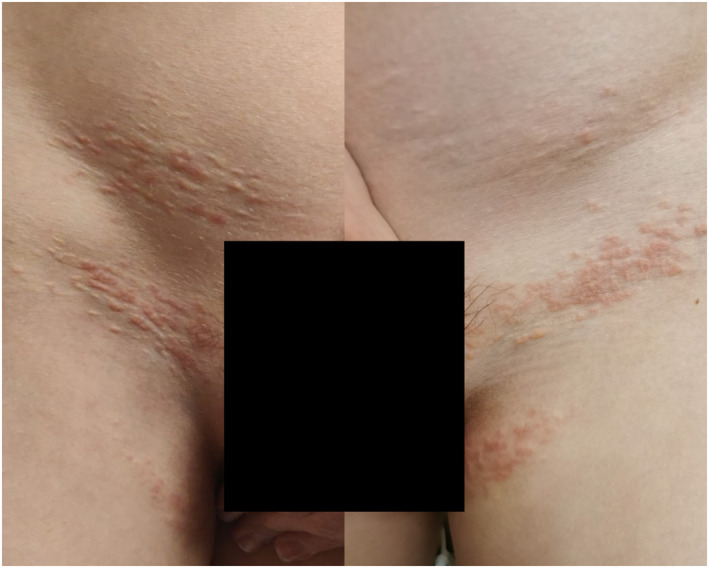
This photograph illustrates the strict localization of erythema on yellowish pseudoxanthoma elasticum papules in the patient.

The patient was a 13‐year‐old French Caucasian with unremarkable medical history except uncomplicated PXE. He had recently experienced several episodes of erythema on his PXE inguinal changes that lasted a few days (Figure 1). Comparative analyses were performed with a control PXE patient, focusing on skin pathology, immunohistochemistry and RNA‐sequencing (RNA‐Seq). Differentially expressed genes were validated using RT‐qPCR. The control was a 20‐year‐old PXE patient whose biological samples were in our biological database and who had never experienced erythematous flares. Skin biopsies were performed on a recent erythematous inguinal PXE lesion in the patient and on the lesional forearm skin in the control. Methods were identical for both patients. Detailed methodology is available in Supporting Information S1. Written informed consent was obtained from the patient's legal guardian. Our study was approved by local ethics committees as part of our PXE phenotypical study (ClinicalTrials.gov, #NCT01446380).

## 3 RESULTS

Skin pathology of the case revealed intense mid‐dermal elastorrhexis surrounded by immune cell infiltrates mainly composed of perivascular T cells and CD163+ histiocytes. T cells expressed cytotoxic markers. There were no inflammation hallmarks in the control, but only classical elastorrhexis.

RT‐qPCR revealed significantly elevated levels of IL6 and TNF‐α messenger RNA (mRNA) in the patient, reflecting an inflammatory environment in the lesional skin. We also observed a high expression of transcripts encoding IL‐12, T‐bet, IFN‐γ and granzyme A in the inflamed skin. In addition, although RNA‐Seq data showed that the expression of IFN‐α1 mRNA was equivalent in both subjects, RT‐qPCR analysis showed an induction in the patient but not in the control. No significant difference was observed for IFNAR and IFN‐β mRNA expression between patients. NLRP2, described as a negative regulator of type 1 IFN production, was downregulated in the patient (RNA‐Seq data). Analysis of activation markers showed higher CD40L expression in the patient, reflecting T cell activation in accordance with an overexpression of the co‐stimulatory molecules CD80 and CD86.

RT‐qPCR and RNA‐Seq showed a strong CCL20 expression in the patient, a chemokine involved in the recruitment of effector/memory T‐cells; the expression of the transcript encoding CCR6, a CCL20 receptor, was not significantly different between patients. Results also revealed that the expression of CCL5 and CXCL8 mRNA was increased in the patient, indicating that immune cell trafficking occurred in the skin of the patient as opposed to that of the control. Matrix metallopeptidases (MMP)‐9 and ‐13 mRNA, but not MMP‐2 mRNA, were significantly overexpressed in the patient.

## 4 DISCUSSION

The major finding of our study is the identification of a Th1 polarization of dermal lymphocytes associated with elevated expressions of inflammatory cytokines and chemokines, and especially CCL20 and CCL5, in an erythematous flare‐up of PXE lesions.

Inflammation is unlikely to be caused by skin friction. Indeed, erythema was strictly localized to PXE papules with no extension to surrounding unaffected skin. Moreover, friction is unlikely to simultaneously affect both inguinal folds and the hypogastric area. Finally, we have already observed (but not explored) the same erythematous phenomenon on skin areas less prone to friction, such as the anterior aspect of the neck.

Although PXE is considered an ABCC6‐mediated metabolic disease,^1^ these results strongly suggest that an inflammatory process could participate in the pathophysiology of PXE. ABCC6 deficiency alters extracellular phosphate metabolism and adenosine signalling in a variety of tissues and organs.^5^ This dysregulation notably causes a pathological deficit in inorganic pyrophosphate (PPi), enhancing calcification susceptibility. However, many aspects of the pathophysiology deriving from ABCC6 dysfunction are still unexplained.^1^

It is only recently that some evidence of a possible inflammatory component in the development of PXE has surfaced.^2^, ^3^, ^4^, ^6^ Two studies measured cytokines from fasting plasma samples originating from the PXE patients followed in our centre. Omarjee and co‐workers observed elevated levels of circulating IL‐6 in PXE patients compared to healthy controls (0.36 vs. 0.00 pg/mL) but this result did not reach statistical significance,^4^ perhaps due to large data variation and/or insufficient number of samples. Brampton et al. also reported significant increases in plasma IL‐6 levels in both PXE patients and a mouse model.^7^ Our results bring histological and molecular data to support this previously unsuspected aspect of the PXE pathophysiology. Even though our data are derived from a single patient, they are unambiguous.

The coexistence of inflammation and calcification in PXE lesional skin raises an important question: Could this type of inflammatory flareups contribute and/or aggravate the pathological calcification or is it merely consequential to calcification? There are some elements of an answer. PXE skin lesions appear to develop during the first two decades of life^6^ and indeed our patient is an early teenager. Furthermore, 11% of surveyed patients report erythema superimposed on affected skin that often precedes an extension of the lesions.^6^ Proinflammatory Th1 lymphocytes are also more active in MMP secretion than naive equivalents^8^ which is consistent with our data showing elevated expressions of MMP‐9 and ‐13 mRNA. This would create conditions favourable to elastic fibre degradation. Note that MMP‐mediated elastin degradation correlates with elastic fibre calcification.^9^ Furthermore, the presence of elevated expressions of MMP‐9 and ‐13 likely leads to the generation of matrikines which could participate/sustain a Th1‐dependent pro‐inflammatory process.^8^ There is now a large body of literature describing inflammation as a key factor that activates calcification. Immune cells, such as pro‐inflammatory macrophages, are key players inducing the formation of hydroxyapatite crystals.^10^ Kauffenstein et al. reported that ABCC6 deficiency in both mouse and humans leads to altered extracellular purinergic signalling, which are potent modulators of immune cell functions during inflammation.^5^

A single patient represents a significant limitation of our study. Unfortunately, we were unable to investigate other patients, despite the fact that our referral centre has a cohort of over 250 PXE patients. Indeed, although erythematous flareups are frequently reported by patients,^6^ they are difficult to characterize due to their transient nature. This prevents biological sampling during scheduled examinations for patients who may live hundreds of kilometres away.

## 5 CONCLUSION

Together with previous literature, our data suggest that inflammation could contribute to the pathology either as an active process or an aggravating factor. If follow‐up studies with more patients confirm our results, it will undoubtedly open new venues for therapeutic possibilities for this rare disease to complement treatments currently evaluated.

## CONFLICT OF INTEREST STATEMENT

None to declare.

## AUTHOR CONTRIBUTIONS

**Samuel Rocour**: Data curation (equal); investigation (equal); validation (equal); visualization (equal); writing – original draft (lead); writing – review & editing (lead). **Emeline Vinatier**: Conceptualization (equal); data curation (equal); investigation (equal); methodology (equal); supervision (equal); validation (equal); visualization (equal); writing – original draft (equal); writing – review & editing (equal). **Céline Fassot**: Conceptualization (equal); investigation (equal); methodology (equal); validation (equal); writing – review & editing (equal). **Jonathan Dauvé**: Data curation (equal); formal analysis (equal). **Agnès Toutain**: Data curation (equal); formal analysis (equal). **Sabrina Fronteau**: Data curation (equal); formal analysis (equal). **Marine Monnier**: Data curation (equal); formal analysis (equal). **Laurence Preisser**: Data curation (equal); formal analysis (equal). **Anne Croué**: Data curation (equal); formal analysis (equal); investigation (equal). **Olivier Le Saux**: Validation (equal); visualization (equal); writing – original draft (equal); writing – review & editing (equal). **Alain Morel**: Validation (equal); visualization (equal). **Yves Delneste**: Conceptualization (equal); data curation (equal); formal analysis (equal); validation (equal). **Ludovic Martin**: Conceptualization (lead); investigation (equal); methodology (lead); project administration (equal); supervision (equal); validation (equal); visualization (equal); writing – review & editing (equal).

## ETHICS STATEMENT

Our study was approved by local ethics committees as part of our PXE phenotypical study (ClinicalTrials.gov, #NCT01446380).

## PATIENT CONSENT

We obtained written informed consent from the patient and his legal guardian for skin biopsy and subsequent analyses.

## Supporting information

Supporting Information S1

## Data Availability

The data underlying this article will be shared on reasonable request to the corresponding author.
