# Supplementary material for: T‐cell‐related skin inflammatory flareups with Th1 polarity in a patient with pseudoxanthoma elasticum
Source: Skin Health Dis. 2024 Nov 26;4(6):e430. doi: 10.1002/ski2.430 (PMC11608869; doi:10.1002/ski2.430)
Supplement: Supplementary file 1 — Supporting Information S1 [file SKI2-4-e430-s001.docx]

**Methods**
***Skin Biopsies***
Incisional skin biopsies were performed on a recent erythematous inguinal PXE lesion in the case patient and on a PXE papule on the forearm in the control patient. After sampling, skin biopsies were either immediately immersed in 4%-buffered formalin or in 1.5ml of RNAlater (Qiagen) then stored at -20°C until RNA extraction.

***Pathology and immunohistochemistry***
Standard staining with HPS (Hemalun-Phloxin-Saffron), as well as orcein and Von Kossa, were routinely performed on 4-μm-thick formalin-fixed paraffin-embedded sections. Immunochemistry was executed using Leica Bond-max autostainer (Leica Biosystems) with the following antibodies: anti-CD3 (clone: LN10, dilution: PAE, Leica), anti-CD4 (clone : SP-35, dilution: 1/10, Cell Marque), anti-CD8 (clone: C8/144B, dilution: 1/160, Dako), anti-Granzyme B (clone: GrB-7, dilution: RTU, Vitro S.A.), anti-CD56 (clone: CD564, dilution: PAE, Leica), anti-CD163 (clone: mono 10D6, dilution: 1/200, Leica).

***RNA analyses***
RNA Extraction: Skin specimens were mechanically homogenized in QIAzol lysis reagent (Qiagen), and the aqueous phase was removed after centrifugation through a Phase-Lock Gel column. Total RNA was purified with RNeasy Mini kit (Qiagen) and eluted into 30μl of RNase-free water. The quantity of extracted RNA was measured with Nanodrop. The integrity of total RNA was assessed by agarose gel electrophoresis.
RNAseq: RNA libraries were prepared with the SureSelectXT RNA Direct Library Preparation kit and the SureSelectXT Human All Exon V6 + UTR probes from Agilent. All libraries were sequenced on an Illumina NextSeq550 in paired-end mode (2 x 75bp) with a target depth of 20 million fragments per sample. Sequenced reads were trimmed with fastp v0.20.1 and mapped to GRCh38 using HISAT2 v2.1.0 both with default parameters. Reads overlapping genomic features were counted with featureCounts v2.0.0 from the Subread package and Ensembl v99. Only uniquely mapped and not duplicated reads were counted. Multiple overlaps of unique genomic feature were not counted.
RT-qPCR: RNA was reverse-transcribed with the Superscript II reverse transcriptase (Life Technologies). Levels of mRNA encoding the indicated proteins were evaluated by RT-qPCR on a LC480 (Roche Diagnostic) using SYBR® Green I Master mix (Thermo Fischer Scientific). Relative quantification was performed with the method developed by Vandesompele et al., using RPS18, TBP, GAPDH and RPL13A as references. The calculation method is based on the conversion of the linear Cq values into a logarithmic scale using the efficiency of PCR as an exponential function. The geometric mean of selected housekeeping genes is used as a normalization factor, allowing eliminating inter-sample variations. The value 1 is given to the calibrator with the highest expression level within a series; the levels of expression of the other genes are then calculated compared to the calibrator.

***Statistical analysis***
Differential expression was conducted using R v3.6.2 and edgeR v3.28.0. Gene Ontology and KEGG pathway enrichment were performed with gage package v1.64.0. Gene Set Enrichment Analysis (GSEA) was performed with CAMERA module of edgeR package v3.28.0 and MSigDB collection v7.1. As only one patient sample was available, a non-replicate analysis was performed. EdgeR is a suitable tool for this task. Accordingly, all differentially expressed genes of interest were validated by RT-qPCR with a p value threshold of 0.005.
